# Impact of hospital internships on success in university summative objective structured clinical examinations: Large-scale experience in a French medical school

**DOI:** 10.1371/journal.pone.0302427

**Published:** 2024-06-13

**Authors:** Florent Carsuzaa, Guillaume Larid, Mickaël Martin, Rémi Coudroy, Maxime Vallée, Marc Paccalin, Kévin Brunet, Etienne-Marie Jutant, for the Poitiers OSCEs group

**Affiliations:** 1 Service d’oto-Rhino-Laryngologie et Chirurgie Cervico-Faciale, Centre Hospitalier Universitaire de Poitiers, Laboratoire Inflammation Tissus Epithéliaux et Cytokines, LITEC UR15560, Université de Poitiers, Poitiers, France; 2 Service de Rhumatologie, Centre Hospitalier Universitaire de Poitiers, Laboratoire Inflammation Tissus Epithéliaux et Cytokines, LITEC UR15560, Université de Poitiers, Poitiers, France; 3 Service de Médecine Interne, Centre Hospitalier Universitaire de Poitiers, INSERM U1313, IRATI Research Group, Université de Poitiers, Poitiers, France; 4 Médecine Intensive et Réanimation, Centre Hospitalier Universitaire de Poitiers, INSERM CIC 1402, IS-ALIVE Research Group, Université de Poitiers, Poitiers, France; 5 Service d’urologie, Centre Hospitalier Universitaire de Poitiers, Université de Poitiers, Poitiers, France; 6 Service de Gériatrie, Centre Hospitalier Universitaire de Poitiers, Université de Poitiers, Poitiers, France; 7 Service de Mycologie et Parasitologie, Centre Hospitalier Universitaire de Poitiers, Université de Poitiers, Poitiers, France; 8 Respiratory Department, Centre Hospitalier Universitaire de Poitiers, INSERM CIC 1402, IS-ALIVE Research Group, University of Poitiers, Poitiers, France; Bilawal Medical College, Liaquat University of Medical and Health Sciences, PAKISTAN

## Abstract

**Background:**

Objective structured clinical examinations (OSCEs) are extensively used in many medical schools worldwide with the stated objective to assess students’ clinical skills acquired during internships. The objective of the present study was to assess the factors associated with success in university summative OSCEs, especially the impact of previous hospital internships in corresponding disciplines and supervision during internships.

**Methods:**

This was a cross-sectional study assessing the results in the summative OSCEs of 4^th^ year medical students during the 2021–2022 academic year in a French medical school. The summative OSCEs included five stations for each student. Each student answered a survey at the end of summative OSCEs about previous internships, the supervision they had and perceived difficulty levels for each station. The scores in each station were assessed according to previous hospital internships in the corresponding discipline. Analysis of predictive factors of success in OSCEs, defined by a score ≥ 10/20 at each station, were performed using univariate and multivariate logistic regression.

**Results:**

Out of the 220 students participating in the summative OSCEs, 182 (83%) answered the survey. One hundred and forty-four (79%) of these students had carried out hospital internships in at least one of the disciplines evaluated during the OSCEs. Students having completed an internship in the corresponding discipline had significantly higher OSCEs scores for interrogation, communication, therapeutic education and procedure stations compared to those who had not. Previous internship in corresponding disciplines was independently associated with success in OSCEs in interrogation (OR 9.45 [1.34–66.8] p = 0.02), clinical examination (OR 6.93 [1.88–25.57] p = 0.004, and therapeutic education (OR 3.09 [1.22–7.82] p = 0.02) stations.

**Conclusion:**

Previous hospital internships in the discipline evaluated by the OSCEs are associated with success in summative OSCEs. This reinforces the importance of student involvement during their hospital internships.

## Introduction

To date, objective structured clinical examinations (OSCEs) are extensively used in many medical schools worldwide and are being progressively developed in France [1, 2]. Up until recently, assessments of medical students in France were based only on a written exam with multiple choice questions, which do not assess students’ clinical skills or prepare them for clinical practice [3]. To address this issue, a reform of the 2^nd^ cycle of medical studies in France in 2021 introduced OSCEs in the assessment of French medical students, with the first nationwide OSCEs planned for 2024 [4]. The results of the national summative OSCEs will be part of the results used to classify students at the end of their 6th year and consequently enable them to choose their specialty. It will increase the discriminatory capacity of current evaluation modalities in French medical schools [5] insofar as it has been shown to be an efficient tool to assess the clinical performance of medical students [6]. The stated objective of the reform is to assess students’ clinical skills acquired during hospital internships in view of enhancing their investment in the latter and of better preparing them for their future clinical practice. Indeed, from the 4^th^ to the 6^th^ year of medical studies in France, medical students spend 50% of their time on placements in hospital departments, generally for periods of 3 months, where they provide clinical follow-up of patients, work in teams with senior doctors and gain enhanced clinical experience in the discipline concerned. Training and supervision during these internships are not standardized, and their impact on success in university OSCEs has not been studied in France or in the international literature. Identification of factors associated with success to the OSCEs is aimed at developing pedagogical avenues to improve student preparation.

The objective of this study was to assess the factors associated with success in summative university OSCEs, and more particularly the impact of previous hospital internships in corresponding disciplines.

## Methods

This was a cross-sectional study aimed at assessing the relationship between the results of the summative OSCEs of 4^th^ year medical students (2021–2022 academic year) in the French medical school of Poitiers university and the results of a survey on OSCEs and previous hospital internships collected at the end of these OSCEs, and the scores obtained for the final faculty tests throughout the same academic year.

### Organization of the summative OSCEs

**Fig 1:**
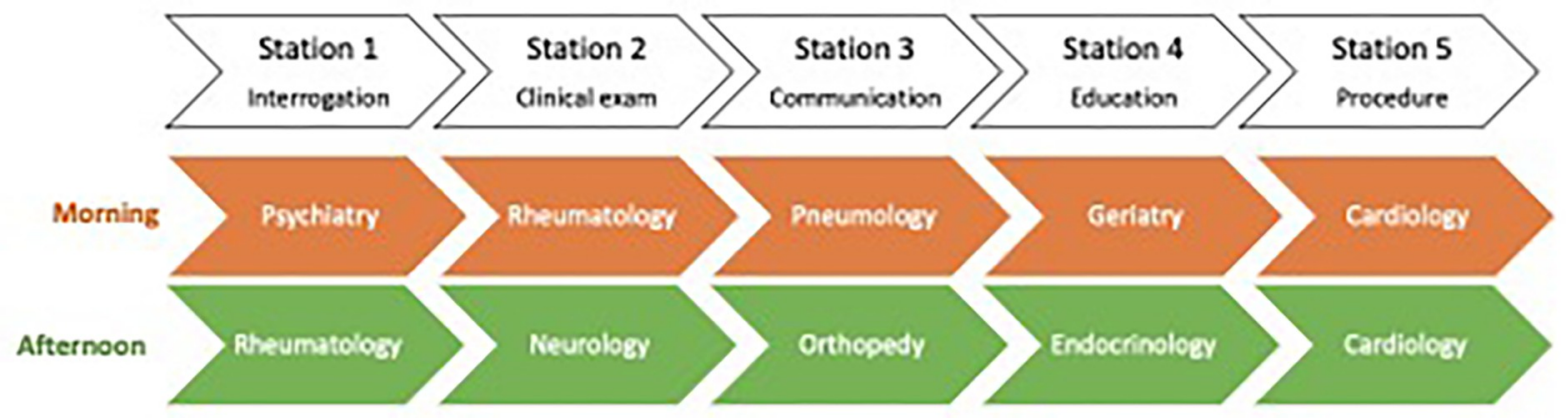
Evaluated organization of summative university OSCEs.

The summative OSCEs took place on May, 31^th^ 2022 for 220 4^th^ year medical students and included a route of five stations for each, randomly assessing disciplines they studied during the academic year. The OSCE disciplines and subjects differed between the morning and the afternoon, evaluating similar skills, the objective being to avoid communication of the subjects between students, with balanced overall difficulty between the two half-days (**Fig 1**).

Simulated patients, trained beforehand so as to ensure evaluation homogeneity, were present at the interrogation, clinical examination, and therapeutic education stations. Each station lasted 7 minutes, during which the students read and followed the instructions. A scoring checklist validated by the educational committee was completed by two evaluators present in the room, with a final OSCE grade of 20. The two evaluators were asked to carry out their evaluation each separately. At the end of the OSCE station, they were asked to use the 2-minute transition time to reach a consensus by reviewing the differences between each evaluation grid. The consensus score was entered into the computer system. The score was normalized according to the number of items evaluated. OSCE grades were collected after each student’s OSCE.

### Survey on OSCEs and previous internships

At the end of the OSCEs, students were asked to answer a survey collecting the following information: places of hospital internships carried out during the 3^rd^ and 4^th^ years of medical studies and whether they had made formative or summative OSCEs for each internship, whether they had performed clinical examinations supervised by a senior, and whether they had participated in consultations in an out-patient clinic. Perceived difficulty level for each station using the Likert scale (0–5) was also collected.

### Ethics

This study was carried-out in accordance with French regulations. The approval of an ethics committee or the consent of the participants was not required as this study was not involving the Human person according to the French Public Health Code (Loi Jardé—n°2012–300 of March the 5th 2012, in application in November 2016—Article R1121-1: research conducted exclusively from the processing of personal data is outside the scope of the researches involving human subjects (RIPH)). The survey was validated by the data protection delegation of the University of Poitiers (n°2022.75). All the data were self-reported by the participants with their written agreement on the questionnaire. Students were free to respond and individuals who did not participate had the same learning opportunities, and their exams assessed in the same way as those who did not participate. The assessment was independent of grading and pseudonymised with the student number. Confidential information was provided as defined by the European General Data Protection Regulation.

### Statistical analysis

Categorical variables were expressed as frequencies and percentages and continuous variables as means and standard deviations. Categorical variables were compared using the chi-square test and continuous variables using the Student’s t-test. Pearson correlation matrix was used to evaluate the relationship between variables. Success at a OSCEs was defined by a score ≥10/20, the minimum score required for validation of summative OSCEs. OSCEs results were analyzed according to the discipline, or the clinical competency assessed. Analysis of predictive factors of success in OSCEs was performed through univariate and multivariate logistic regression with a backward selection of included variables employing a threshold of p<0.20. Included variables were previous internship in the discipline (in 3^rd^ or 4^th^ medical year), activities in the internships (supervised clinical examination participation in consultations, OSCEs), perceived difficulty of the station, final faculty test results in the discipline, and gender. The significance threshold was set at p<0.05. All statistical analyses were performed using GraphPad Prism software (v. 9, GraphPad Software, Boston, USA) and MedCalc (v.20.013; MedCalc Software, Ostend, Belgium).

## Results

### Population characteristics and previous internships

Out of the 220 students, 182 (83%) answered the survey and were included in the study, of whom 112 (61.5%) were women. Thirty-eight (20.9%) had not carried out internships in the disciplines evaluated during their OSCEs, while 71 (39.0%) had carried out an internship in one of the disciplines evaluated, 52 (28.6%) in 2 different disciplines and 20 (11.0%) in 3 disciplines. Medical activities during hospital internships are reported in **Table 1**. Among the 182 students, whatever the discipline, 87 (47.8%) had previously performed ≥1 formative OSCE, 145 (79.7%) ≥1 summative OSCE, 112 (61.5%) supervised clinical examinations, while 144 (84.6%) had participated in consultations during their internships.

**Table 1:** Number of student participants in internships in disciplines evaluated during the university OSCEs.

	Internships, n (% of all sutdents)	Formative OSCEs, n (% of students in the internship)	Summative OSCEs n (% of students in the internship)	Supervised clinical exams n (% of students in the internship)	Consultations n (% of students in the internship)
Cardiology	45 (24.7)	4 (8.9)	16 (35.6)	6 (13.3)	2 (4.4)
Endocrinology	24 (13.2)	3 (12.5)	7 (29.1)	2 (8.3)	5 (20.1)
Geriatrics	36 (19.8)	7 (19.4)	12 (33.3)	8 (22.2)	2 (9.1)
Neurology	30 (16.5)	7 (23.3)	10 (33.3)	7 (23.3)	5 (16.7)
Orthopedy	29 (15.9)	3 (10.3)	5 (17.2)	1 (3.4)	4 (13.8)
Pneumology	18 (9.9)	6 (33.3)	3 (16.7)	5 (27.8)	3 (16.7)
Psychiatry	20 (11.0)	2 (10.0)	6 (31.5)	3 (15.0)	3 (15.0)
Rheumatology	37 (20.3)	8 (21.6)	15 (40.5)	3 (8.1)	5 (13.5)

All the values are expressed in n (%)

### Performances during the OSCEs

The overall average OSCEs score was 11.3 ± 2.0 /20. Procedure and therapeutic education OSCEs were respectively the most (14.6/20 ± 3.2) and the least (8.6/20 ± 3.3) successful. The scores obtained in each station are detailed in **Table 2**.

**Table 2:** Scores for each station.

	n = 182
Overall score (0–20)	11.3 ± 2.00
Station 1: Interrogation (psychiatry)	12.99 ± 3.46
Station 2: Interrogation (rheumatology)	8.9 ± 3.72
Station 3: Clinical examination (rheumatology)	9.21 ± 2.87
Station 4: Clinical examination (neurology)	10.7 ± 3.03
Station 5: Communication (orthopedics)	11 ± 2.77
Station 6: Communication (pneumology)	13.9 ± 2.69
Station 7: Therapeutic education (geriatrics)	10 ± 2.61
Station 8: Therapeutic education (endocrinology)	7.3 ± 3.35
Station 9: Procedure (cardiology)	15.1 ± 2.73
Station 10: Procedure (cardiology)	14.1 ± 3.47

OSCEs: objective structured clinical examinations

All the values are expressed in mean ± standard deviation

### Impact on OSCEs scores of prior internships in a given discipline

**Fig 2:**
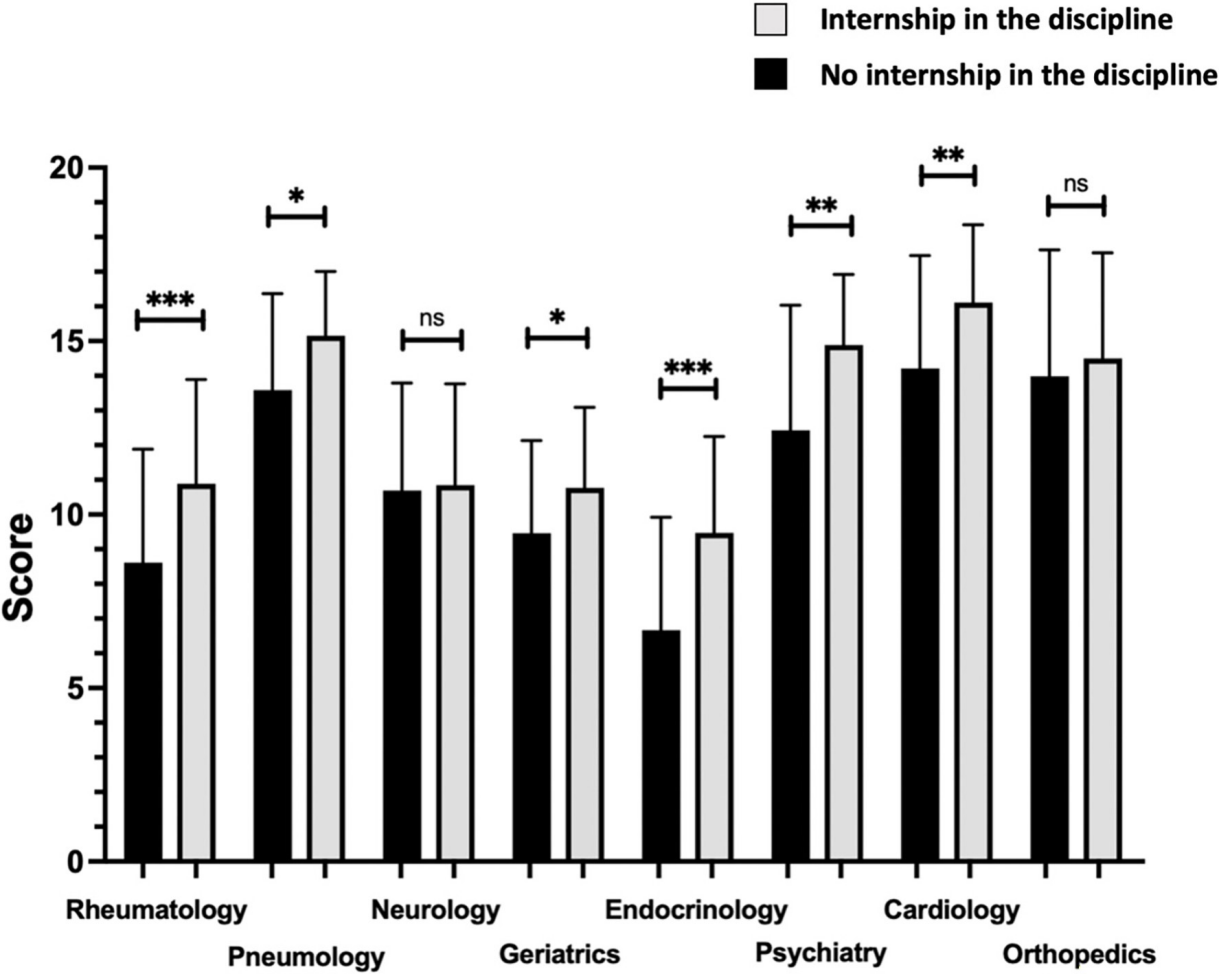
OCSE sub-scores by discipline according to completion of an internship in the corresponding discipline. OSCEs: objective structured clinical examinations. **p<0.01, ***p<0.001, ****p<0.0001.

The scores obtained in the rheumatology (p = 0.0002), pneumology (p = 0.0294), geriatrics (p = 0.0175), endocrinology (p = 0.0004), psychiatry (p = 0.004) and cardiology (0.0011) were higher if the student had completed an internship in the discipline (**Fig 2**).

When OSCEs stations were grouped by evaluated competency, students having completed an internship in the corresponding discipline had significantly higher respective OSCEs scores compared to those who had not done so, in the interrogation (p = 0.0003), communication (p = 0.0004), therapeutic education (p<0.0001), and procedures stations (p = 0.0011) (**S1 Fig**).

**Fig 3:**
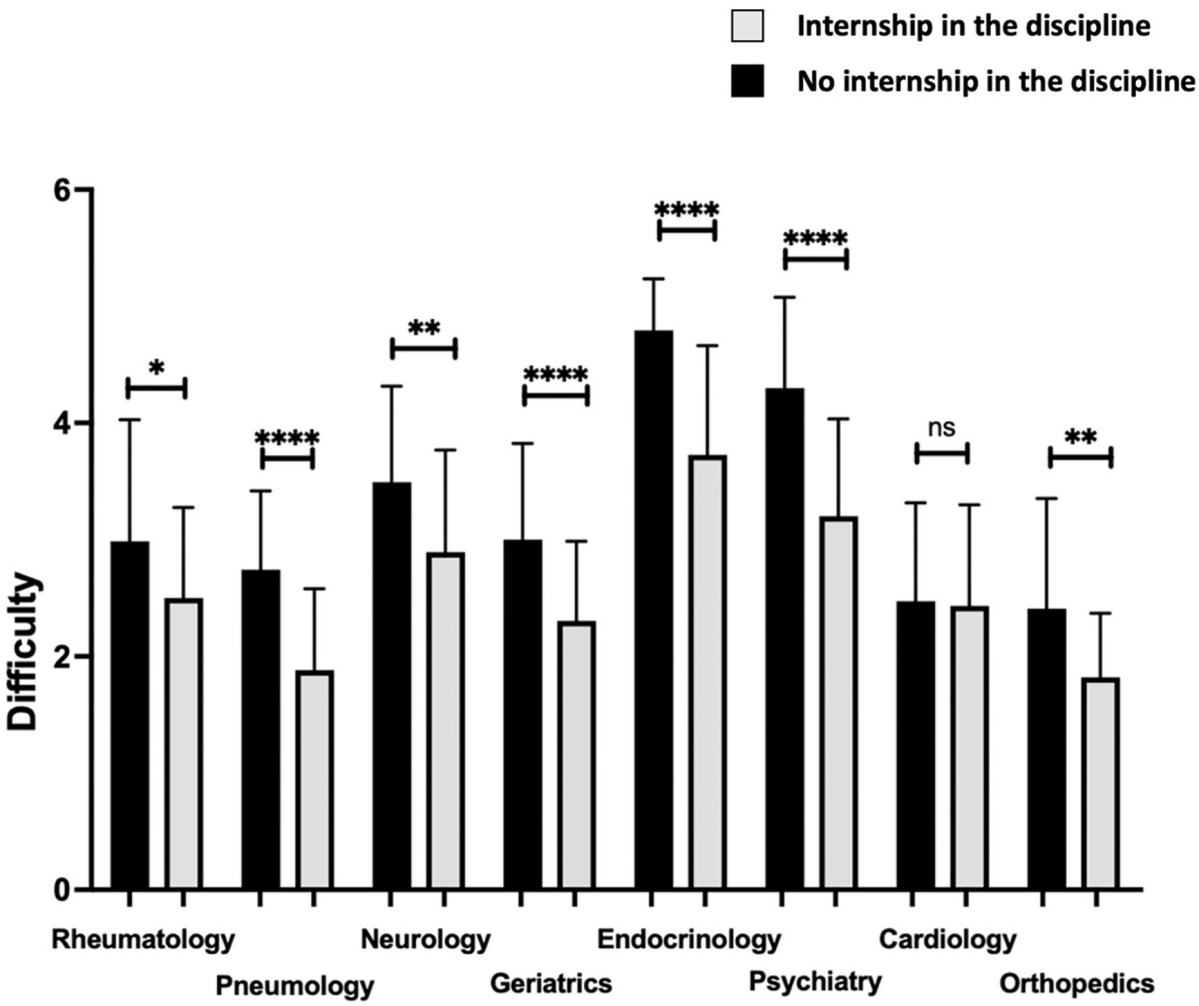
Difficulty level (Likert scale) by discipline according to completion of an internship in the corresponding discipline. **p<0.01, ***p<0.001, ****p<0.0001.

Moreover, the levels of difficulty perceived in the rheumatology (p = 0.0120), pneumology (p<0.0001), neurology (p = 0.0023), geriatrics (p<0.0001), endocrinology (p<0.0001), psychiatry (p<0.0001) and orthopedics (p = 0.0022) units were significantly lower when the student had completed an internship in the discipline (**Fig 3**).

Similarly, when OSCEs station were grouped by evaluated competency, the difficulty levels perceived by students having completed an internship in the corresponding discipline were lower compared to those who had not done so, in the interrogation (p = 0.0004), communication (p<0.0001), and therapeutic education stations (p<0.0001) (**S2 Fig**).

There was an inverse correlation between the difficulty level perceived by the students and the scores obtained in the interrogation (p = 0.0307), communication (p = 0.0025), therapeutic education (p<0.0001) and procedure (p = 0.0063) domains. Conversely, students having performed previous formative or summative OSCEs during internships, whatever the internship, did not have significantly different overall OSCEs scores compared to those who had not done so (11.1 ± 1.9 *vs*. 11.5 ± 2.1, p = 0.16 and 11.4 ± 2.1 *vs*. 11.1 ± 1.67, p = 0.47 respectively). The same results were observed for each clinical domain.

### Performance in the OSCEs and faculty test results

There was a positive and strong correlation between overall OSCEs scores and overall faculty test results throughout the same academic year for each student (r = 0.7639, p<0.0001). Moreover, there was a positive correlation between the faculty test results in the discipline and the corresponding OSCEs score for orthopedics (communication) (r = 0.3772, p = 0.009), endocrinology (therapeutic education) (r = 0.3655, p = 0.0004), cardiology (procedures) (r = 0.3448, p = 0.0012), geriatrics (therapeutic education) (r = 0.3197, p = 0.0032), neurology (clinical examination) (r = 0.2659, p = 0.0108), rheumatology (clinical examination and interrogation) (r = 0.2486, p = 0.0009) and psychiatry (interrogation) (r = 0.2167, p = 0.0464).

### Predictive factors of OSCE success

Univariate analysis of predictive factors of success in OSCEs (score ≥ 10/20) for each station and each discipline are reported in **S1 and S2 Tables.** In multivariate analysis, success in OSCEs was associated with previous internship in the corresponding discipline for the interrogation, clinical examination and therapeutic education stations (OR = 9.45 [1.34–66.80], p = 0.0244 OR = 6.93 [1.88–25.57], p = 0.0036 and OR = 3.09 [1.22–7.82], p = 0.0169 respectively) (**Table 3**). The predictive factors of success to OSCEs stations for each discipline are reported in **Table 4.**

**Table 3:** Predictive factors of success in OSCE stations (multivariate analysis).

	Clinical domain of OSCEs				
	Interrogation	Clinical examination	Procedure	Communication	Therapeutic education
	OR [95% CI]	OR [95% CI]	OR [95% CI]	OR [95% CI]	OR [95% CI]
	p	p	p	p	p
**Previous internship in the discipline**	**9.45 [1.34–66.80]**	**6.93 [1.88–25.57]**	ND	ND	**3.09 [1.22–7.82]**
	**0.0244**	**0.0036**			**0.0169**
**Previous supervised clinical examination**	ND	0.18 [0.02–1.89]	ND	ND	ND
		0.15			
**Previous participation in consultation**	ND	ND	ND	ND	ND
**Previous OSCE**	**0.07 [0.01–0.71]**	ND	ND	ND	ND
	**0.0239**				
**Perceived difficulty level**	ND	0.73 [0.48–1.11]	0.46 [0.19–1.13]	**0.22 [0.10–0.46]**	**0.32 [0.17–0.63]**
		0.14	0.09	**0.0001**	**0.0008**
**Male sex**	ND	ND	0.26 [0.06–1.11]	ND	ND
			0.0685		
**Stress level**	1.59 [0.96–2.63]	ND	ND	ND	ND
	0.0726				
**Theoretical faculty scores**	**1.71 [1.35–2.17]**	**1.39 [1.14–1.68]**	ND	ND	ND
	**<0.0001**	**0.0009**			

OSCEs: objective structured clinical examinations, CI: Confidence interval, ND: Not included in the multivariable model

Logistic regression with backward selection of included variables using a threshold of p <0.20

**Table 4:** Predictive factors of success in OSCEs for each discipline (multivariate analysis).

	Discipline of OSCEs							
	Rheumatology	Psychiatry	Cardiology	Pneumology	Geriatrics	Neurology	Orthopedics	Endocrinology
	OR [95% CI]	OR [95% CI]	OR [95% CI]	OR [95% CI]	OR [95% CI]	OR [95% CI]	OR [95% CI]	OR [95% CI]
	P	P	P	P	P	P	P	P
**Previous internship in the discipline**	**4.21 [1.32–13.46]**	ND	ND	ND	ND	6.01 [0.42–86.42]	ND	**6.92 [1.48–32.46]**
	**0.0152**					0.1879		**0.0141**
**Previous supervised clinical examination**	ND	ND	ND	ND	ND	ND	ND	ND
**Previous participation in consultation**	ND	ND	ND	ND	ND	ND	ND	ND
**Previous OSCE**	ND	0.10 [0.08–1.28]	ND	ND	ND	0.06 [0.002–1.95]	ND	ND
		0.0765				0.1139		
**Perceived difficulty level**	0.71 [0.43–1.18]	ND	0.46 [0.19–1.13]	0.30 [0.06–1.43]	0.52 [0.24–1.12]	**0.20 [0.07–0.53]**	ND	0.23 [0.05–1.09]
	0.1846		0.0899	0.1315	0.0939	**0.0012**		0.0638
**Male sex**	ND	ND	0.26 [0.06–1.11]	ND	ND	0.24 [0.05–1.20]	ND	ND
			0.0685			0.0818		
**Stress level**	ND	**3.66 [1.20–11.17]**	ND	ND	ND	0.54 [0.23–1.28]	ND	ND
		**0.0228**				0.1601		
**Theoretical faculty scores**	**1.45 [1.15–1.82]**	1.57 [0.97–2.55]	ND	ND	ND	1.36 [0,97–1.91]	ND	ND
	**0.0015**	0.0668				0.0747		

OSCEs: objective structured clinical examinations, CI: Confidence interval, ND: Not included in the multivariable model

Logistic regression with backward selection of included variables using a threshold of p <0.20

## Discussion

OSCEs have become an established clinical assessment tool in many countries worldwide and have been developed more recently in French medical studies. Our study is one of the first in Europe and worldwide to study the impact of hospital internships for success in summative university OSCEs. It shows that OSCEs grades are better and the level of perceived difficulties lower when students have carried out an internship in the corresponding discipline. Previous internships were an independent factor of success in OSCEs in interrogation, clinical examination, and therapeutic education stations.

### Positive impact of prior internships on OSCEs scores

The relationship between better results in OSCEs and lower perceived difficulty in the station if previous internship was done in the discipline has previously been described for psychiatry with higher OSCEs scores in the event of a specialized internship [7]. Indeed, the mental status examination, phenomenology, and differential diagnosis are probably more easily apprehended by the student and better mastered if they have been exposed to these diagnoses during internships rather than by learning during lessons or in textbooks [7]. In our study, this observation was corroborated for all the clinical domains evaluated, except for OSCEs scores in clinical examination stations. The two disciplines evaluated in the latter were neurology and rheumatology, and the correlation was significant in the latter, but not in the former. Neurology is taught at an earlier stage of studies and is favored by students as an important [8] but difficult discipline [9]. In France, medical students carry out several full-time 3-month hospital internships in a given discipline, during which they receive clinical training and carry out, independently and under direct supervision, clinical examinations every day. Daily training with actual patients concretely prepares students for clinical practice, and their learning and practice of fundamental skills can help to explain its being a major success factor in completed specialized internships. This finding is supported by a study which shows that OSCEs efficiently assess the clinical performance of medical students [6].

### Lack of impact of formative and summative OSCEs sessions during internships on OSCEs score

Surprisingly, OSCEs success was not associated with completion of the formative and summative OSCEs sessions organized during the various internships, even though OSCEs have been systematized in most hospital internships. This may be explained by differences and lack of homogeneity in the conduct of OSCEs during internships, which are quite new in France, which means that the relevant training of teachers as well as students has remained heterogeneous, and consequently does not yet conclusively determine success in summative university OSCEs.

### Positive correlation between OSCEs scores and faculty test results

In a study assessing the predictive factors for OSCEs success among 5^th^-year medical students, female gender, absence of health problems during the internship, high number of internships in a medical specialty (≥6) and low number of internships in a surgical specialty (<3) were the most significantly factors associated with higher OSCEs success. In this study, success in theoretical faculty exams had no effect [10]. By contrast, in our cohort one of the factors of success in OSCEs was success in final faculty exams. On this subject, a correlation between faculty test results and OSCEs performance was clearly demonstrated in the musculoskeletal domain in a study by the Mexican Board of Rheumatology on postgraduate certification procedures [11]. However, this correlation is not perfect, especially insofar as theoretical faculty exams and OSCEs do not assess the same skills. Another recent study showed a weak correlation between OSCEs grades and faculty exams among 6^th^ year medical students, but these latter obtained lower scores in OSCEs exams compared to other standard evaluation modalities, leading the authors to suggest that the OSCEs are not redundant with the other evaluation modalities [12].

### Better results in procedural skills

The scores obtained in the clinical examination and therapeutic education stations were lower than those obtained in the procedure stations. Therapeutic education requires communication skills, which have been shown to be correlated with OSCEs performance evaluating clinical examination, problem solution prescription, and medical records [13]. On one hand, performance of some procedures (the electrocardiogram…) are part of the daily lives of medical students. On the other hand, communication with the patient, therapeutic education, and performance of specialized clinical examinations, one example being examination of the knee in rheumatology, are skills acquired by only a minority of students. This is in line with a study showing that among the different skills evaluated by OSCEs, medical students are less effective at clinical reasoning skills, while better results are found in procedural skills [14]. It is therefore appropriate to increase this form of skill acquisition in view of upgrading OSCEs results. The particularity of the musculoskeletal examination was previously described in the literature. In this domain, the addition of an interactive workshop with a focus on clinical examination of the knee in the rheumatology rotation of medicine residents led to significantly improved OSCEs performances [15].

Homogenization of internships during medical training courses and relevant activities in the different disciplines help to improve skills acquired by medical students and are likely to improve grades obtained during the OSCEs. Having completed a specialized internship improves success, and the benefit of this continuous training has been underlined in the literature and should be encouraged [16–18].

### Limitations

Our study has several limitations. First, it was conducted at a single institution. However, there was a high response rate (83.1%), which strengthened the results. Moreover, a survey is associated with potential information biases and a certain number of missing data, and result should be considered with caution. In these OSCEs, we used a checklist to determine the student’s grade, but there were no global evaluation scales, which would have improved assessment according to previous internships, as previously shown [19]. In addition, for each station, the two evaluators made a consensus for the final score, and we had no individual scores to assess reliability and generalizability.

## Conclusion

One of the main factors associated with OSCEs success was participation to previous hospital internships in the discipline evaluated by the OSCEs. Homogenization and increased supervised medical activities carried out during the internship will render them even more educational.

## Supporting information

S1 Fig OSCE sub-score by evaluated competency according to completion of an internship in the corresponding discipline. **p<0.01, ***p<0.001, ****p<0.0001. (JPG)

S2 Fig Difficulty level (Likert scale) by evaluated competency according to completion of an internship in the corresponding discipline. **p<0.01, ***p<0.001, ****p<0.0001.
(JPG)

S1 Table Predictive factors of success to OSCE stations (univariate analysis). (DOCX)

S2 Table Predictive factors of success to OSCEs for each discipline (univariate analysis). (DOCX)

S1 Data Minimal data set. (XLSX)
