# Supplementary figures and images for: Impact of hospital internships on success in university summative objective structured clinical examinations: Large-scale experience in a French medical school
Source: PLoS One. 2024 Jun 13;19(6):e0302427. doi: 10.1371/journal.pone.0302427 (PMC11175433; doi:10.1371/journal.pone.0302427)

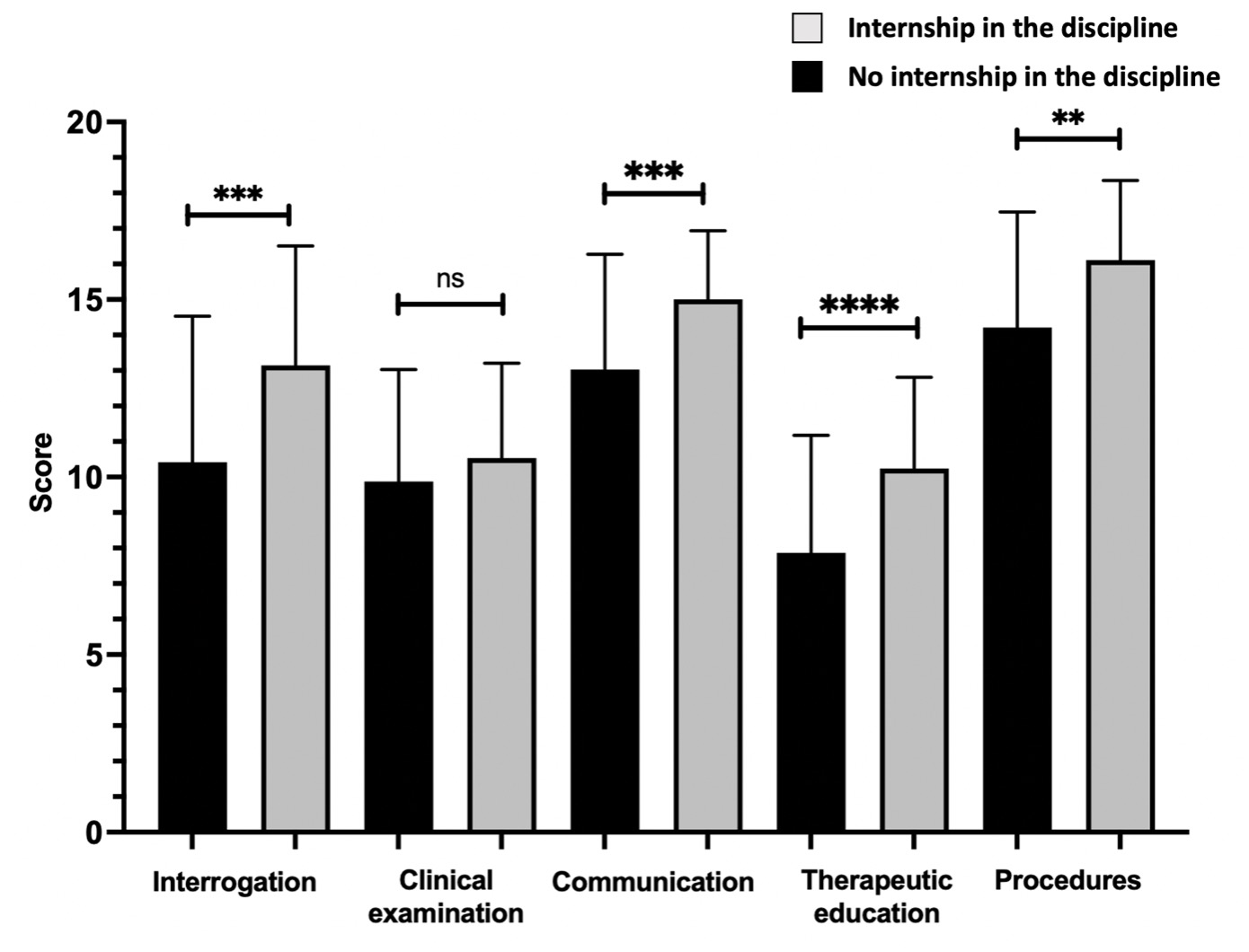

Supplement: S1 Fig — (JPG) [file pone.0302427.s001.jpg]

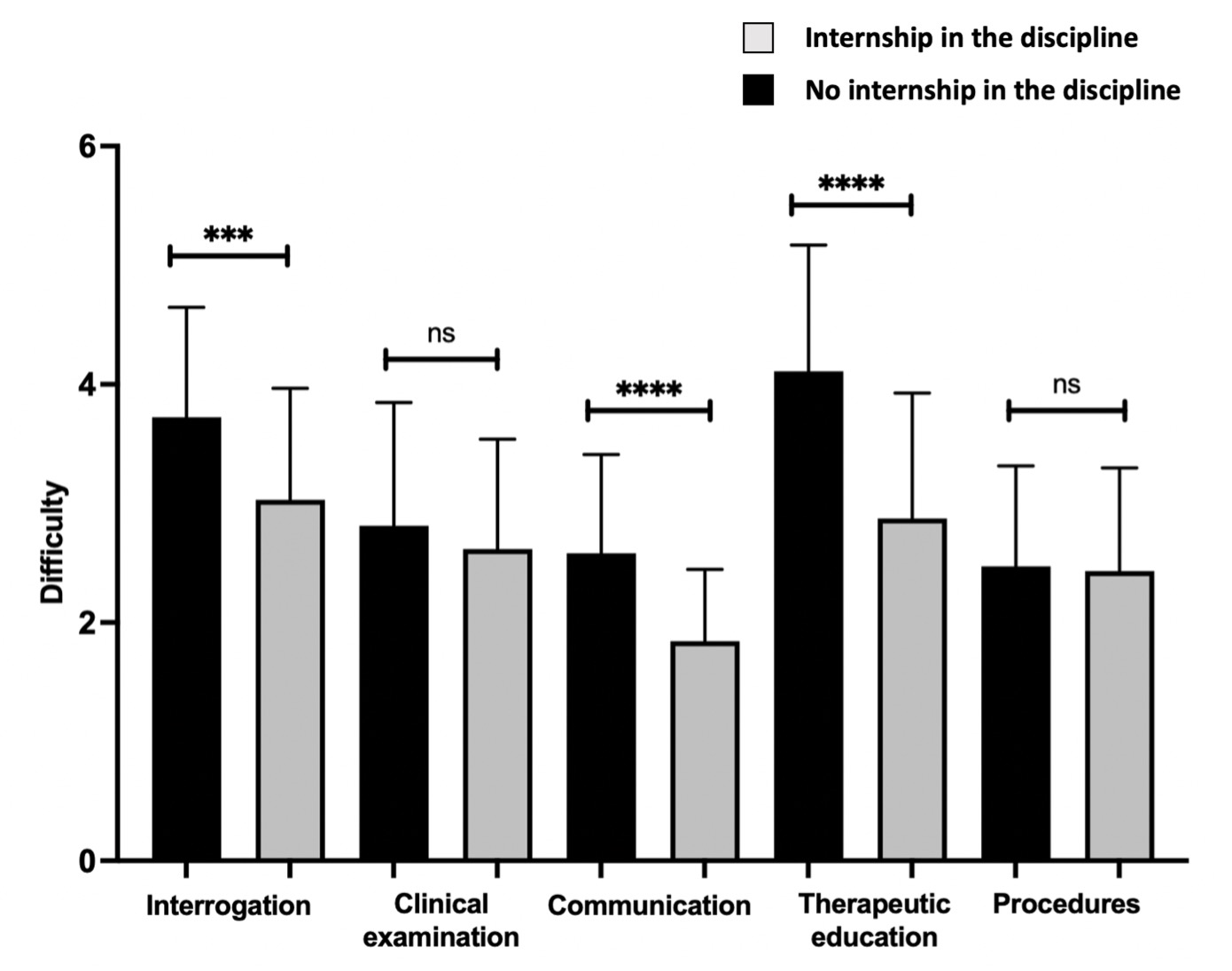

Supplement: S2 Fig — **p<0.01, ***p<0.001, ****p<0.0001. (JPG) [file pone.0302427.s002.jpg]
