# Supplementary material for: Impact of hospital internships on success in university summative objective structured clinical examinations: Large-scale experience in a French medical school
Source: PLoS One. 2024 Jun 13;19(6):e0302427. doi: 10.1371/journal.pone.0302427 (PMC11175433; doi:10.1371/journal.pone.0302427)
Supplement: S1 Table — (DOCX) [file pone.0302427.s003.docx]

**S1 Table. Predictive factors of success to OSCE stations (univariate analysis)**

|  | **Clinical domain of OSCEs** | | | | |
| --- | --- | --- | --- | --- | --- |
|  | **Interrogation**  **OR [95% CI]**  **p** | **Clinical examination**  **OR [95% CI]**  **p** | **Procedure**  **OR [95% CI]**  **p** | **Communication**  **OR [95% CI]**  **p** | **Therapeutic education**  **OR [95% CI]**  **p** |
|  |  |  |  |  |  |
| **Previous supervised clinical examination** | 0.7255 (0.699-5.2688)  P = 0.7511 | 0.7724 (0.2005-2.9752)  p = 0.7074 | NC | 0.3836 (0.6670-2.1960)  P = 0.2818 | 2.6129 (0.7099-9.6178)  P = 0.1486 |
| **Previous participation in consultation** | 1.099 (0.1791-6.7436)  p = 0.9188 | 0.5727 (0.1327-2.4718)  P= 0.4550 | NC | NC | 4.3651 (0.8227-23.1611)  P = 0.0835 |
| **Previous OSCE** | 0.8694 (0.2553-2.9611)  P = 0.8229 | 1.1818 (0.4990-2.7987)  P = 0.7041 | 1.7415 (0.2164-14.0120)  P = 0.6021 | 2.5217 (0.3154-20.6640)  P = 0.3832 | 2.4109 (0.9928 – 5.8546)  P = 0.0519 |
| **Perceived difficulty level** | **1.7854 (1.2819-2.4865)**  **P= 0.0006** | 0.0546 (0.6412-1.1390)  P = 0.2837 | **0.5484 (0.3111-0.9666)**  **P = 0.0377** | **0.2170 (0.1205-0.3909)**  **P < 0.0001** | **0.4363 (0.2933-0.6490)**  **P < 0.0001** |
| **Male sex** | 0.8636 (0.4690-1.1903)  P= 0.6379 | 0.58532 (0.3002-1.0194)  P = 0.0561 | 0.7689 (0.2725-2.1698)  P = 0.6196 | 0.7126 (0.3190-1.596)  P = 0.4085 | 0.8306 (0.4441-1.5535)  P = 0.5612 |
| **Previous internship in the discipline** | **3.2867 (1.4065-7.6801)**  **P = 0.0060** | **2.0829 (1.0491-4.1355)**  **P = 0.0360** | 4.5354(0.5816-35.3698)  P=0.1491 | 0.9004 (0.3697-2.1934)  P = 0.8174 | **1.9756 (1.0342 – 3.7739)**  **P = 0.0392** |
| **Stress level** | 1.1379 (0.7856-1.6484)  P= 0.4943 | 1.0456 (0.7231-1.5119)  P = 0.8126 | 0.7436 (0.3756-1.4724)  P = 0.3954 | 0.8009 (0.4816-1.3319à  P = 0.3922 | 0.8960 (0.6149 – 1.3057)  P = 0.5676 |
| **Theoretical faculty scores** | **1.3378 (1.1731-1.5256)**  **P < 0.0001** | **1.2751 (1.1126-1.4612)**  **P= 0.005** | 0.9917 (0.8396-1.1714)  P = 0.9218 | 0.9074 (0.7426-1.1087)  P = 0.3417 | 1.0477 (0.9150-1.1996)  P = 0.4999 |

OSCEs: objective structured clinical examinations, CI: Confidence interval,

NC: not computable - Logistic regression
