# Supplementary material for: Impact of hospital internships on success in university summative objective structured clinical examinations: Large-scale experience in a French medical school
Source: PLoS One. 2024 Jun 13;19(6):e0302427. doi: 10.1371/journal.pone.0302427 (PMC11175433; doi:10.1371/journal.pone.0302427)
Supplement: S2 Table — (DOCX) [file pone.0302427.s004.docx]

**S2 Table. Predictive factors of success to OSCEs for each discipline (univariate analysis)**

|  | **Discipline of OSCEs** | | | | | | | |
| --- | --- | --- | --- | --- | --- | --- | --- | --- |
|  | **Rheumatology**  **OR** [**95% CI]**  **P** | **Psychiatry**  **OR** [**95% CI]**  **P** | **Cardiology**  **OR** [**95% CI]**  **P** | **Pneumology**  **OR** [**95% CI]**  **P** | **Geriatrics**  **OR** [**95% CI]**  **P** | **Neurology**  **OR** [**95% CI]**  **P** | **Orthopedics**  **OR** [**95% CI]**  **P** | **Endocrinology**  **OR** [**95% CI]**  **P** |
|  |  |  |  |  |  |  |  |  |
| **Previous supervised clinical examination** | NC | 0.4706 (0.0401-5.5164)  P = 0.5484 | NC | **0.0570 (0.0068-0.4786)**  **P = 0.0083** | 3.0769 (0.5850-16.1825)  P = 0.1845 | 0.8302 (0.1747-3.9459)  P = 0.8150 | NC | NC |
| **Previous participation in consultation** | 0.4030 (0.0441-3.6825)  P = 0.4207 | 0.4706 (0.0401-5.5164)  P = 0.5484 | NC | NC | NC | 0.9444 (0.1500-5.9465)  P= 0.9514 | NC | 5.100 (0.7960-32.6757) p = 0.0856 |
| **Previous OSCE** | 1.7333 (0.6187-4.8563)  P = 0.2953 | 0.3358 (0.0515 – 2.1893)  P = 0.2540 | 1.7415 (0.2164-14.0120)  P = 0.6021 | 0.2597 (0.0243-2.7805)  P = 0.2651 | 1.8500 (0.5653-6.0545)  p = 0.3092 | 0.8163 (0.2580-2.5831)  P = 0.7299 | NC | 2.7368 (0.6677-11.2176)  P = 0.1619 |
| **Perceived difficulty level** | 0.8477 (0.6108-1.1764  P = 0.3229 | 1.5921 (0.8552-2.9639)  P = 0.1425 | **0.5484 (0.3111-0.9666)**  **P = 0.0377** | **0.2376 (0.0646-0.8746)**  **P = 0.0307** | 0.6190 (0.3769-1.0165)  P = 0.0580 | 0.3144 (0.1729-0.5714)  P = 0.0001 | **0.2742 (0.1383-0.5434)**  **P = 0.0002** | **0.3288 (0.1313-0.8233)**  **p = 0.0175** |
| **Male sex** | 0.7496 (0.3995-1.4065)  P = 0.3694 | 0.7061 (0.2430-2.0534)  P = 0.5232 | 0.7689 (0.2725-2.1698)  P = 0.6196 | 1.0102 (0.1600-6.3790)  P = 0.9914 | 0.9804 (0.4156-2.3111)  P = 0.9639 | 0.4471 (0.1865-1.0716)  P = 0.0711 | 0.5652 (0.2185-1.4618)  P = 0.2392 | 0.5490 (0.1924-1.5670)  P = 0.2625 |
| **Previous internship in the discipline** | **3.5778 (1.6861-7.5918)**  **P=0.0009** | 2.5962 (0.5402-12.4760)  P = 0.2336 | 4.5354(0.5816-35.3698)  P=0.1491 | **0.1373 (0.0210-0.8990)**  **P = 0.0384** | 1.200 (0.5036-2.8597)  P = 0.6807 | 1.3846 (0.5416-3.5398)  P = 0.4968 | 2.0444 (0.6797-6.1497)  P = 0.2031 | **2.8187 (1.0047-7.9078)**  **P = 0.0490** |
| **Stress level** | 1.1093 (07491-1.6425)  P = 0.6046 | **1.9809 (1.0157-3.8632)**  **P = 0.0449** | 0.7436 (0.3756-1.4724)  P = 0.3954 | NC | 0.9641 (0.5782-1.6076)  P = 0.8885 | 0.8260 (0.4634-1.4725)  P = 0.5170 | 1.2181 (0.6745-2.1997)  P = 0.5130 | 1.0719 (0.5486-2.0946)  P = 0.8389 |
| **Theoretical faculty scores** | **1.2589 (1.0842-1.4618)**  **P = 0.0025** | 1.0841 (0.8624 – 1.3628)  P = 0.4890 | 0.9917 (0.8396-1.1714)  P = 0.9218 | 0.9179 (0.6083-1.3850)  P = 0.6831 | 1.2111 (0.9661-1.5183)  P = 0.0967 | 1.1758 (0.9721-1.4222)  P = 0.0952 | 0.9517 (0.7319-1.2376)  P= 0.7120 | 1.0496 (0.8589-1.2826)  P = 0.6360 |

OSCEs: objective structured clinical examinations, CI: Confidence interval,

Logistic regression
